# Distinct 3D Architecture and Dynamics of the Human HtrA2(Omi) Protease and Its Mutated Variants

**DOI:** 10.1371/journal.pone.0161526

**Published:** 2016-08-29

**Authors:** Artur Gieldon, Dorota Zurawa-Janicka, Miroslaw Jarzab, Tomasz Wenta, Przemyslaw Golik, Grzegorz Dubin, Barbara Lipinska, Jerzy Ciarkowski

**Affiliations:** 1 Faculty of Chemistry, University of Gdansk, Wita Stwosza 63, 80-308, Gdansk, Poland; 2 Department of Biochemistry, Faculty of Biology, University of Gdansk, 80-308, Gdansk, Poland; 3 Faculty of Biochemistry, Biophysics and Biotechnology, Jagiellonian University, Gronostajowa 7, 30-387, Krakow, Poland; 4 Malopolska Centre of Biotechnology, ul. Gronostajowa 7a, 30-387, Krakow, Poland; National University of Singapore, SINGAPORE

## Abstract

HtrA2(Omi) protease controls protein quality in mitochondria and plays a major role in apoptosis. Its HtrA2^S306A^ mutant (with the catalytic serine routinely disabled for an X-ray study to avoid self-degradation) is a homotrimer whose subunits contain the serine protease domain (PD) and the regulatory PDZ domain. In the inactive state, a tight interdomain interface limits penetration of both PDZ-activating ligands and PD substrates into their respective target sites. We successfully crystalized HtrA2^V226K/S306A^, whose active counterpart HtrA2^V226K^ has had higher proteolytic activity, suggesting higher propensity to opening the PD-PDZ interface than that of the wild type HtrA2. Yet, the crystal structure revealed the HtrA2^V226K/S306A^ architecture typical of the inactive protein. To get a consistent interpretation of crystallographic data in the light of kinetic results, we employed molecular dynamics (MD). V325D inactivating mutant was used as a reference. Our simulations demonstrated that upon binding of a specific peptide ligand NH_2_-GWTMFWV-COOH, the PDZ domains open more dynamically in the wild type protease compared to the V226K mutant, whereas the movement is not observed in the V325D mutant. The movement relies on a PDZ *vs*. PD rotation which opens the PD-PDZ interface in a lid-like (budding flower-like in trimer) fashion. The noncovalent hinges A and B are provided by two clusters of interfacing residues, harboring V325D and V226K in the C- and N-terminal PD barrels, respectively. The opening of the subunit interfaces progresses in a sequential manner during the 50 ns MD simulation. In the systems without the ligand only minor PDZ shifts relative to PD are observed, but the interface does not open. Further activation-associated events, e.g. PDZ-L3 positional swap seen in any active HtrA protein (*vs*. HtrA2), were not observed. In summary, this study provides hints on the mechanism of activation of wtHtrA2, the dynamics of the inactive HtrA2^V325D^, but does not allow to explain an increased activity of HtrA2^V226K^.

## Introduction

The human HtrA2 (high-temperature requirement A2) protease controls protein quality in mitochondria [1, 2]. It is involved in cell death (apoptosis, both caspase dependent and independent [3–5]) and consequently plays a role in oncogenesis [5, 6]), and in Parkinson [4, 7, 8] and Alzheimer diseases [9–13]. These properties allow to consider HtrA2 as a potential drug target in cancer [5, 14] and/or neurodegenerative disorders [4].

**Fig 1:**
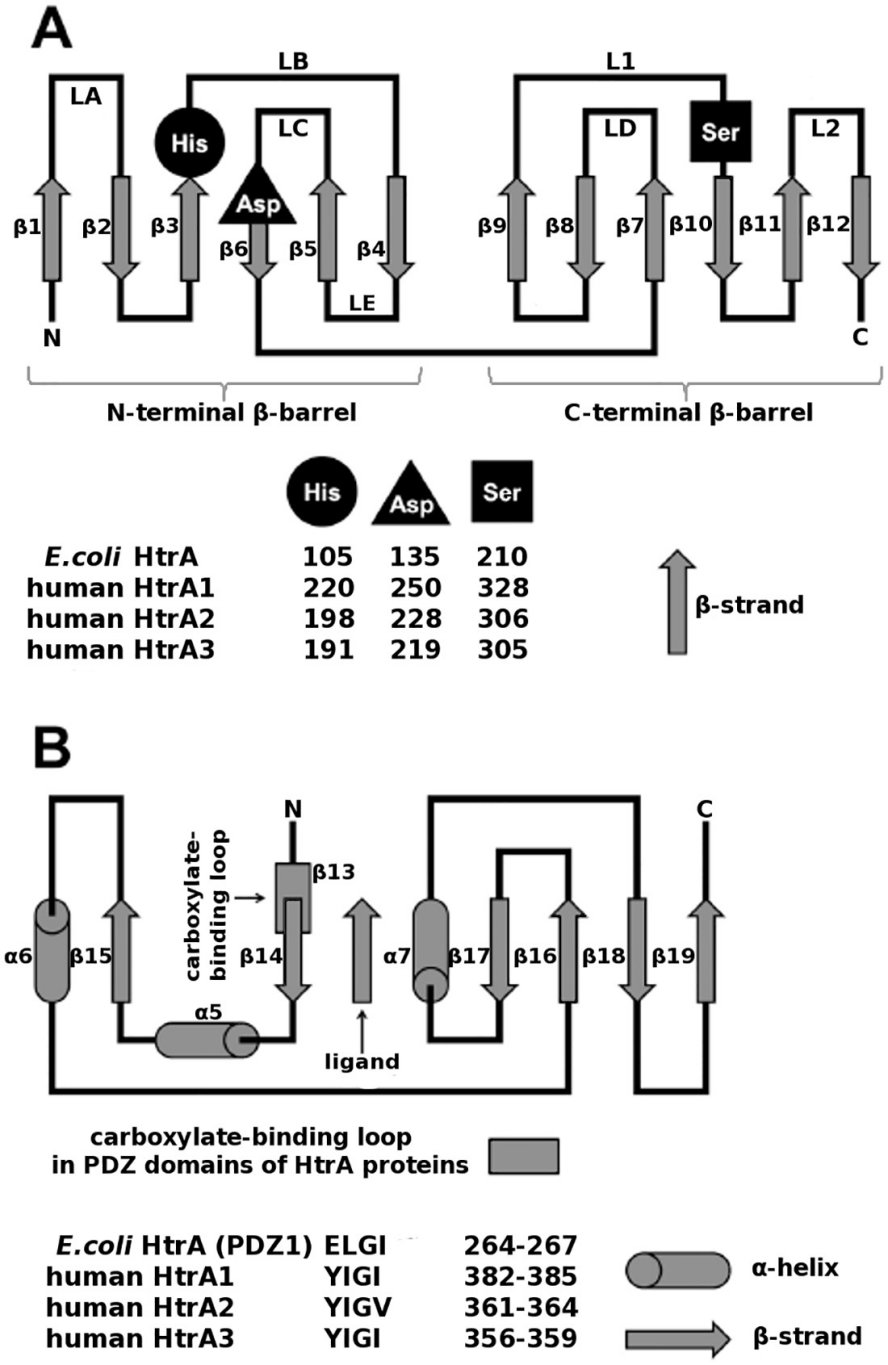
Human HtrA2 topology. (**A**) Protease domain: the N- and C-terminal helices, α1/α2 and α4, respectively, are omitted for clarity. The catalytic triad His, Asp, Ser and the loops are labeled according to the chymotrypsin nomenclature [16]. (**B**) PDZ domain. The carboxylate binding loop is indicated.

The HtrA proteases can be distinguished from other serine proteases by the presence of one or two C-terminal PDZ (Postsynaptic density of 95 kDa, Disk large, Zonula occludens 1) domain(s). The protease domain (PD) of the chymotrypsin type consists of two perpendicularly arranged β-barrel lobes β1-β6 and β7-β12. The lobes are delimited by the N- and C-terminal α-helices, arranged parallel in space, in a C2-pseudosymmetrical manner. These secondary-structure elements are connected by loops, several of which are important for proteolytic activity [15]. The loops are named according to the chymotrypsin nomenclature: LA(β1-β2), LB(β3-β4), LC(β5-β6), LE(β4-β5) in the N-terminal barrel and LD(β7-β8), L1(β9-β10), L2(β11-β12), L3(β8-β9) in the C-terminal one [16] (Fig 1). The active site and the catalytic triad (His, Asp, Ser) are embedded in the interface of the β-barrels.

The PDZ domains participate in the regulation of HtrA activity by recognizing and binding hydrophobic C-terminal sequences of substrates or regulatory peptides. The HtrA monomers form higher-order oligomers. The common structural unit is a pyramid-shaped trimer with PDs constituting the pyramid walls and their N-termini adding to a central core around the pyramid apex, while the PDZ domains protruding outward laterally at the pyramid base. Two bacterial HtrAs, the *E*. *coli* DegP and DegQ proteases, which contain two PDZ domains, further oligomerize forming up to 24-mers. Regardless the oligomerization status, at low temperatures or in the absence of a substrate or activating peptides HtrAs adopt inactive conformations characterized by non-catalytic architecture of the triad and/or a restricted access to the active site. Hence, it is generally accepted that HtrA proteases are activated [15, 17–20] by binding of a specific activator at the PDZ domain and a substrate at the active site.

HtrAs share ~60% sequence homology. A number of crystal structures of pro- and eukaryotic HtrAs have been solved to date [17, 20–34], demonstrating that the enzymes exist in at least two distinct conformations: resting and active states. There are several recent reviews on structure-function relationships within HtrAs [15, 19, 35], including a minireview in the introduction to our previous work [36].

Human HtrA2 has a single PDZ domain and in the crystal structure forms a pyramid-shaped homotrimer. The catalytic triad located in an interior (concave) of the trimer—a feature shared by the whole HtrA proteins family—comprises H198, D228, and S306 (S306A mutation in the crystal [31]) in a noncanonical, catalytically inactive orientation [19]. PDZ and PD are connected by a flexible linker. PDZ contains a typical peptide binding groove between β14 and α7 starting from a peptide-recognition motif ^361^YIGV^364^, the PDZ-specific carboxylate-binding loop β13-β14 [37]. In HtrA2 the groove is buried in the tight interface between PDZ and PD, and so is the PD active site; this being a unique structural feature of this protein, not observed in other family members, letting the trimer compare to a budding flower or—its monomer unit—to a lid-enclosed jug. PDZ packs against PD through van der Waals contacts, involving two clusters of hydrophobic residues in (β11-L2-β12)_PD_-(β14-α5)_PDZ_ (hereafter hinge A) and (β5-LC-β6)_PD_-(β13/α7)_PDZ_ (hinge B). With PDZ taken as a lid to PD, the hinges are located at the C- and N-barrels within the PD domain, respectively [31]. According to Li et al. [31], HtrA2 activation requires an activating peptide to bind at hydrophobic groove of PDZ, which leads to the opening of the PDZ-PD interface and enables protease activity. The model is supported by the fact that PDZ-deleted HtrA2 variant is more active than the full-length protein [31, 38]. Moreover, specific peptides binding to the PDZ domain increase the HtrA2 activity [9, 39]. HtrA2 activity is also up-regulated in response to a heat-shock [40]. Available evidence jointly suggests that activation of HtrA2 may resemble that of DegS [24, 26]. However, while in DegS the PDZ domain only stabilizes the inactive form of the protease, in HtrA2 it physically blocks the access of the substrate to the active site.

Recently, we have investigated temperature-dependent (20–45°C) conformational changes in HtrA2 using single-tryptophan mutants at the PD/PDZ interface and monitoring fluorescence of this residue. Correlation of temperature dependence of fluorescence and catalytic rates in selected mutants allowed us to elucidate the role of particular amino acids in regulation of protease activity [36]. We have concluded that when the HtrA2 structure relaxes and the PDZ-PD interface opens, the PD part of hinge A retains its structural integrity. As such it does not tolerate any mutations in the (β11-L2-β12)_PD_ segment, e.g. V325D, without complete loss of protease activity. On the contrary, hinge B also retains major interactions upon HtrA2 activation, but may accommodate favorably selected mutations, e.g. V226K in LC_PD_, that increase the proteolytic activity of the protein compared to the wild type HtrA2 [36].

In our most recent study we combined a temperature-dependent tryptophan-induced quenching (TrIQ) with selected mutation-activity analyses. TrIQ is a modern technique complementary to FRET and efficient at smaller distances (≤10Å) than the latter [41]. We found that in activation, PDZ changed its position versus PD of own and of the adjacent subunit PD* (the asterisk indicates the adjacent subunit). These changes included (β14-α5)_PDZ_ slightly closing distance to “own” LC and L2 and to LD* [38] while simultaneously increasing distances between all other not contributing to the “hinges” (see above) parts of bulk PDZ and PD, including in particular the regulatory L3 [35, 38] moving away of own PDZ, compare Fig 1 in [38]. Simultaneously, expanding our former structure-activity mutation study [36], we have found that any mutations weakening inter-domain interactions at the PD-PDZ-PD* interfaces increase HtrA2 proteolytic activity, see e.g. Figs 3 and 4 and Table 2 in Ref. [38], including the V226K mutation, see above.

The aim of this study was to examine in details likely routes of HtrA2 activation, compatible with the above observations. We used the wild type HtrA2, the inactivating mutant in hinge A (V325D) and the activating mutant in hinge B (V226K) as models. Our successful crystallization and structure solution of HtrA2^S306A/V226K^ (having the catalytic Ser routinely disabled to avoid self-degradation) revealed that S306/A/V226K mutant has the same (inactive) architecture, earlier found for HtrA2^S306A^ [31]. Subsequent molecular dynamics (MD), both unrestrained and restrained (RMD), have led us to a hypothesis on basic structural requisites to HtrA2 activation, pertinent to the structure relation between: 1) On the one hand, a unique tight PDZ-PD interface in the inactive HtrA2 (see above) that confines the regulatory L3 to a flap on a convex side of the trimer; and 2) On the other, our observation that an active form turns up to be common for the HtrA family [17, 20, 23, 24, 26, 28–30, 32], featured by opened PD-PDZ interface, having L3 slid between L2_PD_ and α7_PDZ_ onto the concave side of the trimer, thus destroying hinge A. Consequently, bringing inactive HtrA2 to this “canonical active architecture” would require an extensive rearrangement of L3, equivalent with a crack of hinge A and replacement it with a new (L2-L3)_PD_- (β13-α7)_PDZ_ interactions, with simultaneous whole opening of the PD-PDZ interface. An alternative would mean an entirely different activated HtrA2 architecture than one typical of all active HtrAs resolved thus far.

## Methods

### Preparation of HtrA2^V226K/S306A^ protein

*E*. *coli* strain BL21(DE3) (Novagen, San Diego, USA) transformed with the pET-derived pDZ5 V226K plasmid [36], carrying *HtrA2*^*V226K/S306A*^ gene was used to overproduce mutant protein (amino acids 134–458) with His_6_-tag at the C-terminal end. The protein was purified by affinity chromatography on Ni-NTA according to the manufacturers' instructions (Qiagen, Wroclaw, Poland). The concentration of HtrA2^V226K/S306A^ was estimated using Amido Black as described before [42]. The purity of the HtrA2^V226K/S306A^ preparation was estimated at more than 95% by SDS-polyacrylamide gel electrophoresis.

### Crystallization, data collection and structure solution

Immediately prior to crystallization screening the buffer was exchanged to 5 mM Tris-HCl pH 8.0 containing 150 mM NaCl and 200 mM imidazole by gel filtration on Superdex s75 (GE Healthcare), the protein was concentrated to 18 mg/ml and screening was performed using sitting drop vapor-diffusion method. Crystals appeared after several days at room temperature in Crystal Screen 2 formulation 21 (Hampton Research). The initial conditions were optimized. The crystals used for measurements were obtained from 0,15 M MES pH 6.5 containing 2 M NaCl, 0,13 mM KH_2_PO_4_ and 0,1 M NaH_2_PO_4_. The crystals were cryoprotected in 25% glycerol in mother liquor and cooled in liquid nitrogen. The diffraction data were collected using in house rotating anode copper source (MicroMax-007 HF; Rigaku). Data were indexed and integrated with MOSFLM [43]. The following steps were performed using software collected in the CCP4 package [44]. Data were scaled with Scala [45, 46]. Molecular replacement was performed using Phaser [47] with alanine search model based on the structure of HtrA2 (PDB ID: 1LCY). Model building was performed manually using Coot [48]. Water molecules were added using Coot and were inspected manually. Restrained refinement was performed with Refmac5 [49]. Throughout the refinement 5% of reflections were used for cross-validation analysis [50] and the behavior of R_free_ was utilized to monitor the refinement strategy. The data collection and refinement statistics are presented in Table 1. The structure was deposited in PDB with the accession code 5FHT.

**Table 1:** Data collection and refinement statistics.

PDB ID	5FHT
Wavelength (Å)	1.54
Resolution range (Å)	14.57–1.95 (2.019–1.95)^a^
Space group	H3
Unit cell	86.01 86.01 126.70; 90.0 90.0 120.0
Total reflections	121190 (16150)
Unique reflections	25396 (3720)
Multiplicity	4.8 (4.3)
Completeness (%)	99.6 (99.5)
Mean I/sigma(I)	7.4 (3.1)
Wilson B-factor	38.41
R-merge	0.116 (0.405)
R-work	0.1749
R-free	0.2275
Number of atoms	2500
macromolecules	2285
water	200
Protein residues	300
RMS(bonds)	0.019
RMS(angles)	1.97
Ramachandran favored (%)	97.9
Ramachandran outliers (%)	0.35
Average B-factor	34.37
macromolecules	33.64
solvent	42.66

^a^Values in parentheses represent the highest-resolution shell.

### Molecular dynamics of the trimers of HtrA2 and its mutants

As differences between the first HtrA2^S306A^ [31] and the current HtrA2^S306A/V226K^ structure (5FHT, this work *vide infra*) have turned out to be negligible, the crystal structure of the former (HtrA2^S306A^, pdb code 1LCY [31]) was used as a template. 1LCY and 5FHT have 3 unresolved regions, viz: the N-terminal IAP-binding motif ^134^AVPSP^138^ and ^134^AVPSPPPA^141^; L3_β8~β9_^282^ARDLGLPQT^290^ and ^281^PARDLG^286^; and the PD~PDZ linker ^344^RGEKKNSSSGISGSQ^358^ and ^345^GEKKNSSSGISGSQ^358^, respectively. We ignored the IAP-binding sequence as one of no importance in protease function [31], while L3 and the PD~PDZ linker structures were restored using the BIOPOLYMER and LOOP SEARCH modules in the SYBYL software [51]. The obtained model was optimized using the minimization protocol implemented in the AMBER11 package [52, 53]. PDZ domain in complex with a selectively-binding peptide was modeled using the crystal structure of the *lone* (i.e. lacking PD) PDZ-GWTMFWV complex (pdb code 2PZD) [54] because the PDZ domains in 1LCY and 2PZD overlapped perfectly (RMS<0.1Å). Several minimizations and short low-temperature MD simulations were performed in repetitive cycles to reduce all steric clash between the introduced peptide and PD and to simultaneously preserve the initial structure as much as possible.

Based on the above modeled structure the V226K and V325D mutants were constructed. Additionally their equivalents with the catalytic triad serine replaced by alanine were also built: S306A, V226K/S306A and V325D/S306A. Each of the latter three was modelled with and without the selective ligand GWTMFWV. All these models were optimized as described above. Homotrimers compatible with 1LCY crystal structure were made from optimized starting monomers by imposing the C_3_ symmetry typical of the 1LCY crystal. Nine constructed starting homotrimers were separately immersed in a rectangular TIP3P [55] water box of 125x125x95 Å^3^ size. To neutralize the negative charge on the protein system, 6, 9 and 12 Na^+^ ions were added to HtrA2^V226K^, HtrA2 and HtrA2^V325D^ trimers (and to the matching S306A mutants), respectively. Each trimer was initially optimized using similar methodology as described for the monomers.

MD simulations for each of the 9 systems were carried out using the following parameters: 1 fs time step, constant pressure (1 atm) and periodic boundary conditions. SHAKE algorithm [56] was used for treatment of hydrogen atoms. Long-range electrostatic interactions were simulated using the Particle Mesh Ewald (PME) method [57] with the cutoff equal to 8Å. Non-bonding interactions were updated every 25 steps. The simulation temperature was set to 313K because it is known that the thermally induced activation of HtrA2 is efficient at this temperature [36, 38]. Each of the simulated systems consisted of about 43,000 water molecules, 3x320 or 3x327 amino acid residues (apo and ligand containing models, respectively), ~145.000 atoms in total. The productive runs were 50 ns long, with snapshots taken every 1 ps. To obtain the results in a reasonable time, all molecular systems were simulated using AMBER PMED v2.2 software [58, 59] and NVIDIA GPU-CUDA [60] card hardware.

### Restrained (steered) molecular dynamics of the HtrA2 monomer

To this aim, the crystal structure of HtrA2^S306A^ (1LCY [31]) monomer was used as a template. The L3 (^282^ARDLGLPQT^290^) and the linker (^344^RGEKKNSSSGISGSQ^358^) gaps in the sequence were preserved. Moreover, by trial-and-error we found crucial to truncate both the β8 inlet to and the β9 outlet from the L3 gap, each by extra 8 residues, ^274^IVSSAQRP^281^ and ^291^NVEYIQTD^298^, respectively. Otherwise both trunks would sterically hinder optimization of the 1LCY-like start structure to a target meeting the restraints deduced from TrIQ and having a more open PD-PDZ interface (see below). All above conditions held, an alternative start, compatible with the completely open (no PD-PDZ1 interface) structure of the active DegP (PDB file 3CS0) [17] was used as a control. Nine PD-PDZ distance restraints were imposed in agreement with Fig 1B,C and Table S3 in Ref. [38]. As TrIQ results provided only tendencies (increase/decrease/keeping of a C^α^-C^α^ distance) in HtrA2 thermal activation [38, 41], we affixed to these tendencies, specific, though arbitrarily guessed restraint distances, see Table 2. The harmonic restraints were very soft, with force constants equal to 1 kcal/Å. To maintain the complete shape integrities within the PD and PDZ domains, the backbone angles (φ,ψ,ω) were fixed. Successful restrained MD was carried out at 45K using AMBER11 [52, 53].

**Table 2:** The restraints used in restrained MD of 1LCY monomer. The values in the middle column are inferences affixed to tendencies given in Fig 1 B,C in Ref. [38].

	Distance, [Å]		
C^α^- C^α^	1LCY [31]	Restraint	Optimized, see Results
I179- L398	7.96	26.00	23.5
A201-Y361	8.44	13.00	14.6
P225-Y361	8.47	13.00	13.0
P225-V364	8.79	13.00	13.6
M323-P384	17.19	25.00	21.2
M323-M365	8.93	9.00	10.7
F331-M365	12.31	12.00	12.1
V226-L367	9.71	8.00	9.5
F331-I373	11.13	10.00	12.0

## Results

### Crystal structure of HtrA2^V226K/S306A^

**Fig 2:**
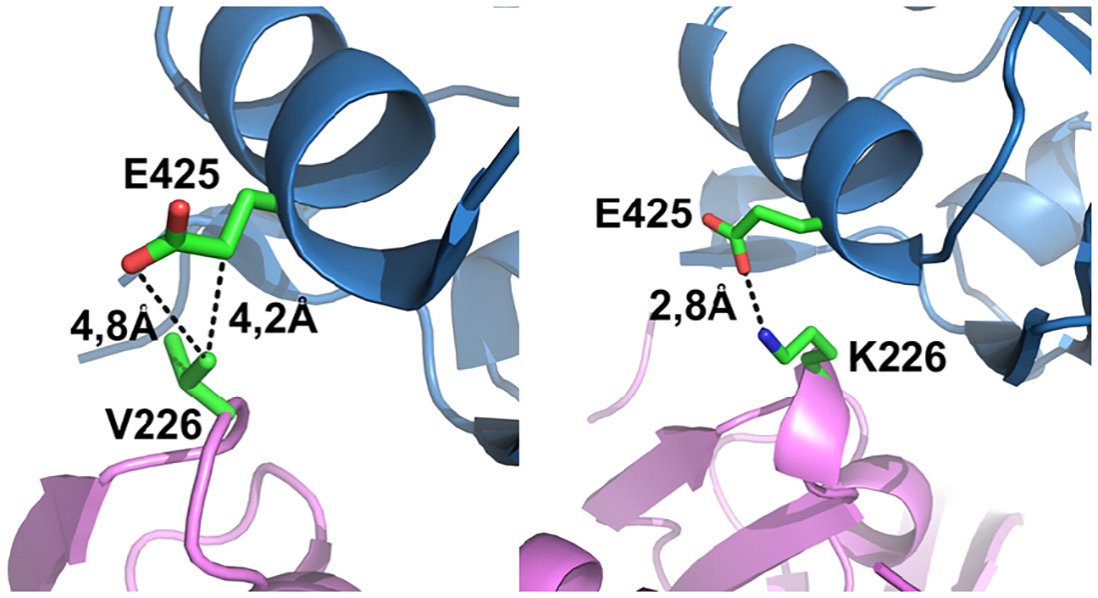
Interdomain interactions in hinge B area. **Left:** HtrA^S306A^ (1LCY) [31] and **Right:** HtrA^V226K/S306A^ (5FHT; this work). Proteolytic domain is colored magenta, PDZ domain—blue. In the V226K mutant (**Right**) the distance 2,8Å indicates an H-bond/ionic pair Lys226_(LC)PD_ -E425_(α7)PDZ_, absent in HtrA2 (**Left**). Otherwise, both structures overlap perfectly on each other, see text.

To analyze the structural consequences of activating mutation V226K in hinge B of HtrA2 we obtained a crystal structure of the mutant HtrA2^V226K/S306A^. Its overall architecture resembles that of HtrA2^S306A^ [31]. No structural rearrangement of PDZ domains vs. PD domains is noted compared to HtrA2^S306A^ (RMS<0.4Å, based on 290 [98%] common Cα atoms). Minor changes are observed only around the mutated residue and on L3. The mutated K226 forms a hydrogen bond with E425, absent in the HtrA2^S306A^, while on L3 the unresolved gap reduces from 9 in HtrA2^S306A^ [31] to only 6 residues ^281^RARDLG^286^, more firmly manifesting L3 to flap on a convex side of the trimer than in 1LCY. The additional hydrogen bond K226-E225 further stabilizes the interaction between PDZ and PD domains in hinge B region (Fig 2). Previous studies have demonstrated that the HtrA2^E425L^ exhibits activity comparable to HtrA2^V226K^ and the activities of both mutants are higher compared to that of the wild type [36]. While V226K mutation creates an additional hydrogen bond adding to the PD-PDZ interaction, the E425L mutation results in hydrophobic interaction of V226 and L425 within the same region of the protein, with a like effect in the inactive structure of HtrA2(Omi).

### Preliminaries of molecular dynamics

**Fig 3:**
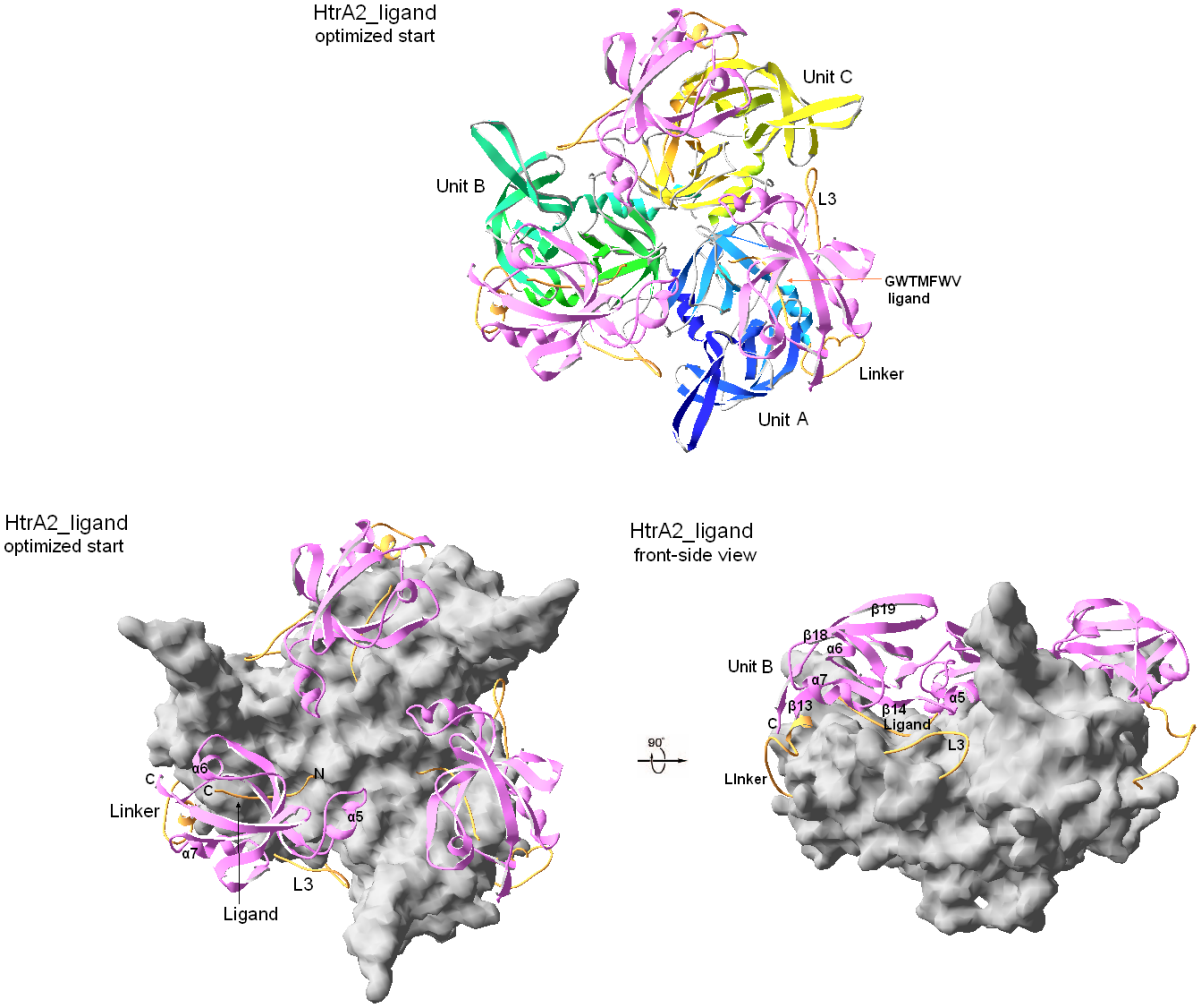
Optimized starting trimer of HtrA2. **Upper panel:** The view perpendicular to the C3 axis onto the concave of the trimer. Units A-C are distinguished by color of protease domain ribbons: blue (A), green (B), yellow (C); the PDZ domains are shown uniformly as pink ribbons. The restored fragments, missing in 1LCY structure: L3, PD-PDZ-linker and the ligand, are highlighted as orange threads (plus short orange helices constituting part of the linkers). **Lower Panel Left:** Orientation as above, PDs are represented by a gray surface. PDZ ribbons (making 3 lids to the PDs) and modeled fragments are colored as above. **Lower Panel Right**. A lateral view of the latter. Selected features in the units are labeled in all views.

Since the gaps in L3 and the PD-PDZ linker, not defined in the crystal structure, were restored computationally (see Materials and Methods), all the models were subject to initial optimization to relax any unfavorable contacts. This optimization only minutely adjusted the PD-PDZ arrangement compared to that seen in the crystal structure (Fig 3). This is evidenced by small values of root-mean-square (RMS) deviation calculated for the positions of C^α^ atoms between the optimized trimers and those found within the crystal structure of HtrA2^S306A^ (1LCY) [31] (Table 3). The most pronounced adjustments concerned: i) unresolved fragments added computationally, including the ligand, and ii) adjustments of monomers at their interfaces within the trimer. None of the adjustment steps exceeded 1 Å of total RMS.

**Table 3:** Modeling statistics of the starting structures.

RMS [Ǻ]: steps i) and ii); (step i) only) [see text]	HtrA2^S306A^ ^b^	HtrA2^S306A^-pep^c^
**1LCY** [31] ^a^	1.7; (0.8)	1.8; (1.0)
**HtrA2**^**S306A**^ ^b^		1.0; (0.9)

^a^ **1LCY**: Cα-based RMS calculation excludes residues unresolved in 1LCY [31] structure.

^b^ **HtrA2**^**S306A**^ exemplifies 3 tested mutants: S306A, S306A/V226K and S306A/V325D, whose optimized starting structures fit to RMS<0.1 Ǻ.

^c^ **HtrA2**^**S306A**^**-pep** exemplifies 6 tested cases containing peptide ligand: S306A, S306A/V226K, S306A/V325D, HtrA2 wild-type, V226K and V325D, whose optimized starting structures fit mutually to RMS<0.1 Ǻ.

Importantly, the ligand-binding and the apo structures have optimized similarly demonstrating the correctness of our approach to modeling the peptide in complex with the apo-HtrA2. The GWTMFWV peptide fits surprisingly well to the apoPD-PDZ interface. Notably, its insertion into 1LCY has introduced only 2 *new* contacts (using the 3.5 Å criterion) with PD, viz. those of W2 with F331(β12) and W6 with A197(LB). Based on the mere geometrical criterion, they appeared as modest as 7 contacts already present in the crystal structure of PDZ-ligand, viz. with (M366,L367)_β14_, E376_α5_, (H394,I397)_β15_ and (A424,Y428)_α7_ [54]. A minor adjustments of W2-T3 in the ligand and of β11-L2-β12 in PD have alleviated these unfavorable interactions. Thus, the ligand pocket of PDZ, even if seemingly inaccessible in resting HtrA2, is spacious enough to accept the heptapeptide.

### Productive molecular dynamics

**Fig 4:**
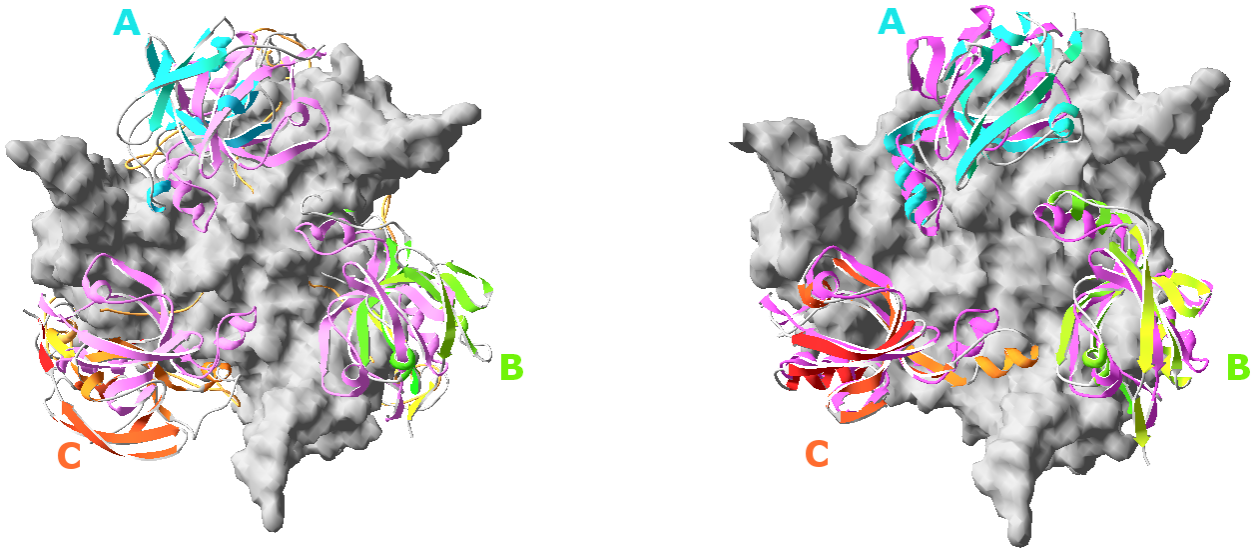
HtrA2 trimers after 50 ns MD, with reference to the starting structures: The pink PDZ (ribbons) make 3 lids to the PD (surface) of the trimer viewed perpendicular to C3 as in Fig 3. **Left:** The resultant **wtHtrA2-ligand** complex. PDZ domains, in Units A, B, C, (cyan, canary and amber ribbons, respectively) have moved equatorially (budding-flower-like) relative to their starting (pink) positons, especially in units A & C thus opening the PD-PDZ interface. **Right:** The resultant **HtrA2**^**S306A/V325D**^ **double mutant-ligand** complex. Moving PDZ domains are colored as in the left panel. Only minor PDZ motions are seen; no opening of the PD-PDZ takes place.

**Fig 5:**
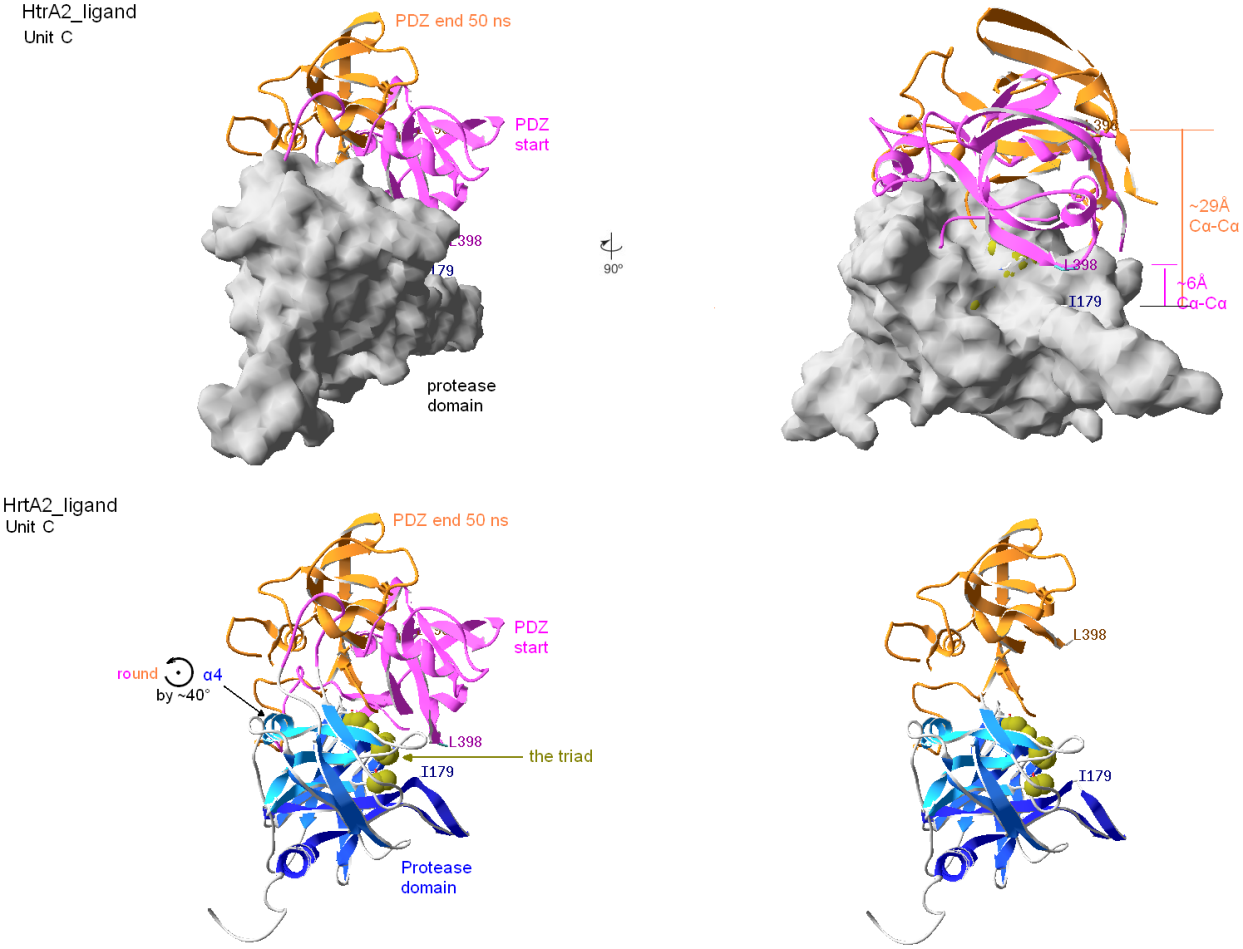
HtrA2-ligand complex unit C after 50 ns MD with reference to the initial structure. All but the top-right views have common orientation. PD N-terminal α1-α2 and C-terminal barrel are in the foreground (bottom, dark blue ribbon), while C-terminal α4 and N-terminal barrel are in the background (bottom, light blue ribbon). α1-α2 and α4 axes run roughly perpendicular to the figure plane. Important features of the structure and its dynamics are indicated. **Top-left**: Unit C as extracted from the trimer in Fig 4 left panel, and reoriented (see above). **Bottom-left**: As above, PD unwrapped form the surface. **Top-right**: Unit C is rotated 90° round the vertical axis to show relationships between the PDZ motion and exposure of the catalytic triad (olive green). **Bottom-right**: Visualization of a true uncovering of the catalytic triad after ca. 40° PDZ counterclockwise rotation (lid-opening) during the 50 ns MD, characterized in detail in the neighbor bottom-left panel and in Table 4.

Within 50 ns MD simulations each system experienced mainly specific segmental/collective motions of PDZ and PD domains relative to each other (Table 4), but retained intra-segmental consistency within the domains. Save for the N-terminal α1 (residues 139–150), β1-LA-β2 (residues 168–177), L3 (residues 282–290) and the PD-PDZ linker (residues 346–352), which have undergone more pronounced rearrangements, inherent drifts of the domains from their original architectures have not been larger (RMS) than 1.2–1.5Å and 1.1–1.3Å for ~100 PDZ and ~160 selected PD C^α^ atoms, respectively. It is important to note that two of the four most dynamic structure elements, L3 (9 residues) and PD-PDZ-linker (15 residues) were those restored algorithmically [51]. Therefore they might have had their starting 3D architectures not soundly consistent with the structural context of the reported HtrA2 trimer and as such expectedly could have exhibited increased mobility to adjust to surrounding structure environments. Therefore, the only significant motions observed in the simulations have included segmental translations/rotations of PDZ relative to PD within each monomer (Figs 4 and 5).

**Table 4:** Overview of simulation results. For a viewer having the N-terminus and PD C-terminal barrel in the foreground, as in Fig 5, “cc” and “c” denote, respectively, counterclockwise and clockwise rotation of PDZ versus PD. The viewer sees this rotation roughly round an axis parallel to α4 and passing for “cc” between α4 and α7, see Fig 5, and; for “c” by the peptide-binding motif ^361^YIGV^364^. “Δ”applies to “cc” only and refers to another measure of extent of the “cc” rotation, viz. to its associated arc at maximized radius. I179(β2) and L398(β15) roughly fit this radius tips, hence their C^α^-C^α^ (vs. ~6Ǻ at the start) distance increases with “cc” rotation defining “Δ“. “i” indicates minute irregular motions.

	Unit A	Unit B	Unit C (Refer to Fig 5)
containing peptide ligand			
HtrA2	cc ~30°/ Δ = 16.4 Ǻ	cc ~0°/Δ = 10.5 Ǻ	cc ~40°/Δ = 23.1Ǻ
HtrA2^S306A^	i	cc ~50°/Δ = 24.4 Ǻ	i
HtrA2^V226K^	c ~10°	i	i
HtrA2^V226K/S306A^	i	i	i
HtrA2^V325D^	i	i	i
HtrA2^V325D/S306A^	i	i	i
no ligand (apo)			
HtrA2^S306A^	i	i	i
HtrA2^V226K/S306A^	i	i	i
HtrA2^V325D/S306A^	i	i	i

The segmental motions observed in the simulated models classify into three basic types (Table 4). The prevailing motion relies on minor and apparently non-specific rearrangements of PDZ versus PD. It is seen in all but one monomers of the HtrA2^S306A^ and HtrA2^V226K^ models, all monomers of HtrA2^V226K/S306A^, HtrA2^V325D^, HtrA2^V325D/S306A^ models in the peptide containing trimers, as well as in the all apo HtrA2^S306A^, HtrA2^V226K/S306A^ and HtrA2^V325D/S306A^ trimers.

Wild type ligand-bound HtrA2 and HtrA2^S306A^ exhibit the second type of motion. With the MD progress two monomers within the former structure and one within the latter have performed a relatively consistent motion, illustrated in Fig 5. For a viewer having the N-terminus and the C-terminal barrel of PD in the foreground, PDZ rotates counterclockwise relative to PD around an axis between and roughly parallel to α4_PD_ and α7_PDZ_, simultaneously passing near the N-front of α5_PDZ_. Some translation, hard to characterize with math rigor (see, however, **Supplementary Material**, containing the Principal Component Analysis, PCA, of the MD results), accompany the rotations of these units. The rotations are equivalent to opening the PD-PDZ interface in a lid-like way. The two hinges (A and B) are made of clusters of hydrophobic residues. The rotation of up to 50° (Table 4 and Fig 5) enables complete access both to the catalytic triad in PD and the peptide-binding site in PDZ. It is surprising that a similar motion is not seen in MD of HtrA2^V226K^, even though this mutant was demonstrated experimentally to exhibit increased proteolytic activity compared to the wild type [36].

The last type of motion, seen only in a single monomer of the ligand-bound HtrA2^V226K^ also lifts the lid, but only slightly, about 10°, clockwise and “another way around”, as if the hinges were now fixed on the opposite side of the PD-PDZ interface. Trajectory analyses using the math rigors of PCA (see Supplementary Material) have confirmed the above conclusions.

Our results demonstrate that the motions within the monomers in the trimer are not synchronous in the timescale of 50 ns (Fig 4). By selecting a pair of residues most remote to the hinges, e.g. PDZ(lid) L398(β15-α6 loop) with PD(jug) I179(β2) we measured the extent of opening of the PD-PDZ interface (Table 4 and Fig 5) in diverse units.

The conformations of the catalytic triad residues were analyzed in the optimized starting and resultant product structures and have been found catalytically incompetent, as seen previously in the crystal structure. Typical distances in a catalytically ready triad in serine proteases of the HtrA type amount to 3.15±0.2 Ǻ for the O^γ^(Ser)-N^ε2^(His) distance and to 3.3±0.4 Ǻ for the N^δ1^(His)-O^δ1^(Asp) distance ([19], compare also Ref. [32]). The average lengths of the sides of a triangle formed by the C^α^-C^α^ distances within the catalytically competent triad, Δ_S-H_ = 8,6±0.1 Ǻ, Δ_D-H_ = 6.5±0.1 Ǻ and Δ_S-D_ = 10.2±0.1 Ǻ) alike serve as a criterion of the catalytic competence of HtrA2. Neither any starting nor resultant structures have fitted these criteria. We speculate that substrate binding induces further rearrangements yielding a proteolytically competent conformation within the catalytic triad, though this is not reflected in our simulations where substrate has not been taken into account.

### Restrained Molecular Dynamics (RMD)

**Fig 6:**
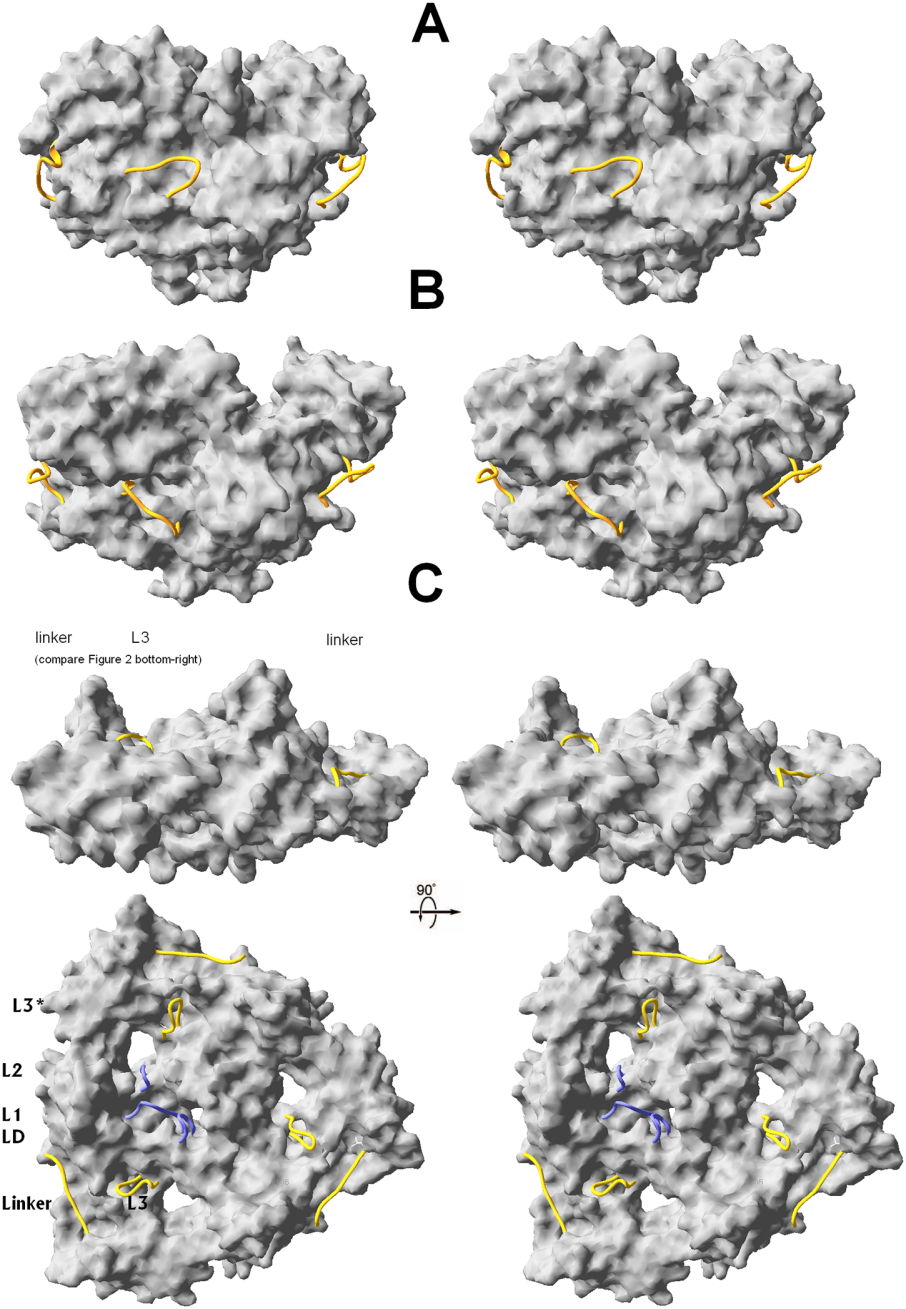
HtrA trimers; PD-PDZ(1) in surface representation. L3 and the linker are shown as orange threads, L2, L1 and LD as blue threads. (**A)** wtHtrA2-peptide starting structure in orientation identical to that in Fig 3 bottom-right. (**B)** wtHtrA2-peptide after 50 ns MD. (**C**) active DegP PDB (entry 3CS0 [17]) PDZ2 is omitted for clarity. In contrast to **A** and **B**, where L3 protrudes outside on the convex side of the trimer, in the active DegP and in other active HtrAs (not shown) L3 enters between PD and PDZ, onto the trimer’s concave, contributing (vide bottom) to the allosteric activation cascade: L3*-LD-L1-L2 [35].

In Figs 5 and 6 of our recent work [38] we have sketched an initial step to thermal activation of HtrA2, complying qualitatively with the TrIQ restraints. The model relies on a *slight* opening of the PD-PDZ(red) interface, in agreement with the MD results given here in Figs 4 and 5. In order to more rigorously resolve the PD-PDZ orientation fulfilling these restraints [38], now we have worked out the TrIQ results using RMD and arrived at the following results (see also Table 2). (i) Satisfying the TrIQ-inferred temperature-driven increases and decreases of selected 9 intra-unit distances is unfeasible, unless the already incomplete L3 is further truncated at each the β8 inlet to, and the β9 outlet from the L3 gap by extra ~8 residues, which removes steric hindrance of L3 to optimization. (ii) The optimization arrives at the same result no matter starting PD-PDZ orientation, i.g. the fully closed 1LCY-like [31] or fully open 3CS0-like, see Methods. (iii) The result, defined by the nine C^α^-C^α^ distances given in the last column of Table 2, features a PD-PDZ opening intermediate between that in Fig 5 this work and Figs 5 and 6 in [38].

## Discussion

The crystal structures of HtrA2^S306A^ [31] and HtrA2^S306A/V226K^ (this work) resolve to homotrimers having essentially identical architectures, characterized by a unique PD-PDZ arrangements not seen in the structures of other procaryotic and eukaryotic proteins of HtrA family. In contrast to the other family members, regardless in proteolytically more or less active forms, the catalytic and the PDZ domains in HtrA2 tightly pack against each other [31]. A major consequence of this feature is an apparent inaccessibility of the activating-peptide binding site on the PDZ part and of the substrate pocket on the PD part in both structures. As such, the structures meet a model of an inactive HtrA2(Omi) protein [31].

In our 50 ns MD simulation, two subunits in the trimers of HtrA2-ligand and one in the trimer of HtrA2^S306A^-ligand (Figs 4 and 5 and S1, S2 and S4 Figs) have reorganized in agreement with an opening of the tight PD-PDZ interface by lid-like rotations of ~30°-50° (Fig 5 and Table 4). The rotation was around an axis ~parallel to and located between α4_PD_ and α7_PDZ_ and passing by hinges A and B (Figs 4 and 5 and S4 Fig). These rotations resemble opening of the inactive HtrA2 toward structures typical of all proteolytically-competent HtrAs determined to date. The motions are in agreement with the FRET result reported by Chaganti et al. [61] because, while releasing access to the PDZ and PD binding sites, they maintain the F341C_α4_-Y428W_α7_ (F208C_α4_-Y296W_α7_ in their terminology) distance in hinge B roughly intact, as concluded from their FRET measurements [61]. These Authors also studied HtrA2 allosteric activation using MD [61–63]. They stipulated that the inactive, closed form of HtrA2 employs a non-canonical binding groove by the PD-PDZ interface, before being able to utilize the canonical peptide-recognition motif [62] i.e. the (β13-β14)_PDZ_ carboxylate-binding loop, see above. In this work we neither pursue this issue nor the allosteric regulation of HtrA2 (caspase-dependent) via its N-terminus [63].

To date, 15 proteolytically active HtrA structures of widely diverse origin have been published, including *E*. *coli* HtrA(DegP) [17], *Mycobacterium tuberculosis* HtrA2 [23], *E*. *coli* DegS [20, 24, 26, 32], *Legionella falloni* DegQ [28], plant *Arabidopsis thaliana* Deg1 [29] and human HtrA1 [30]. We note that their common feature is a similar mutual location (even if diverse orientation) in space of their PDZ (PDZ1 in DegP and DegQ [17, 28]) *vs*. PD domains. Both domains do not form an interface. In the human HtrA1 the PDZ domain is not resolved within the crystal structure due to its high flexibility [30], however, HtrA1 can be active without PDZ domain. Similarly, human HtrA3 [34] and *E*. *coli* DegS [20, 26, 32] do not require PDZ domain for activity. Even more conservative, shared by all structures of the active proteases are the positions of the sensor loop L3. Despite its length varying by up to 6 residues between different HtrA proteases, L3 occupies in all active HtrA proteases a specific site between PD and PDZ domains, where it separates L2_PD_ and α7_PDZ(1)_, simultaneously being in contact with LD* of the proximal unit of the trimer (asterisk denotes a neighboring monomer). In fact, these commonalities apply to all three L3*-LD-L1-L2 “activation clusters” per trimer, mutually related by the C_3_-symmetry (Fig 6C) [15, 19, 35]. On the contrary, in the structure of inactive human HtrA2 and during entire MD simulation carried out for HtrA2- and HtrA2^S306A^-ligand system the sensor loop L3 persists in clearly different site.

**Fig 7:**
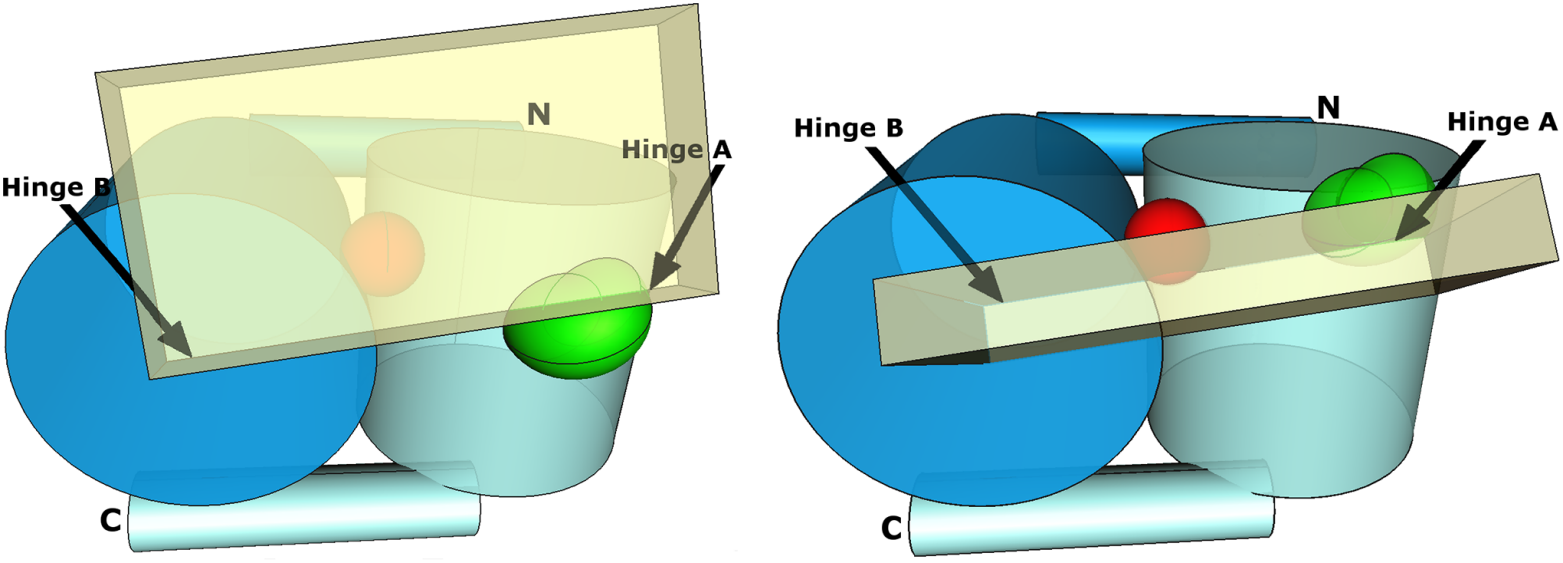
Diagrammatical scheme of structural requisite to HtrA2 proteolytic activation. N-helix and barrel are colored blue, C-barrel and helix are cyan, L3 is green, the triad is red, PDZ domain is pale yellow orthorhombic box. **Left:** Inactive closed form, L3 flaps on the exterior (convex in the trimer) side, opposite the location of the triad. **Right:** Active open form, L3 is slid between C-barrel and PDZ to the interior (concave in the trimer) side, disrupting hinge A.

Fig 6 illustrates that L3 and (β13-α7)_PDZ_ (proximal to the C-barrel side of PD, a part of hinge A) are in HtrA2 (Fig 6A) reversely positioned in space than they are in an active structure of any HtrA protein, taken DegP as an example (Fig 6C). Moreover, this “wrong” arrangement augments with the progress of MD (Fig 6B). None of the subunits exhibiting the PD-PDZ opening motion is capable of letting L3 enter between the own C-barrel and PZD as required to activate the protease. Reversely, the opening motion(s) pushes L3 more equatorial, to the exterior (convex) side of the gradually opening concave of the PD-PDZ interface (Fig 6A and 6B). In a view of the published experimental data on specific contribution of L3 in the activation process, it is clear that the (semi)open HtrA2 units do not approach true active states in MD. To achieve this, L3 and PDZ should have swapped their positions (Fig 6C), a too demanding task for any simple MD. This structural requisite is diagrammatically presented in Fig 7 for a monomeric unit.

It is noteworthy that the HtrA2^V226K/S306A^ mutant (Table 1, entry 5FHT), crystallizing version of HtrA2^V226K^ more active than the wtHtrA2 [36], has crystallized in exactly the same inactive architecture as HtrA2^S306A^ [31], with its L3 even more distinctly flapped out to the exterior (convex side) of the PD-PDZ interface than the latter.

In summary, X-ray study combined with MD failed to explain higher proteolytic activity of the V226K mutant, because in the MD simulations wtHtrA2-ligand complex exhibits more pronounced PD-PDZ opening than HtrA2^V226K^-ligand does, while one would expect the opposite from comparing their activities. At the same time our studies explain the inactive phenotype of V325D mutant, which manifests no tendency to PD-PDZ opening in the MD simulation. Our results, combined with accumulated knowledge about structures of activated HtrAs has led to structural requirements, drawn schematically in Fig 7, that have to be met on a path from inactive to proteolytically active HtrA2(Omi) protease. While opening the PD-PDZ interface, somewhere on a way to activation an L3-PDZ positional swap is required, which could only be attained upon a crack of hinge A, the (β11-L2-β12)_PD_-(β14-α5)_PDZ_ cluster, while retaining hinge B, the (β5-LC-β6)_PD_-(β13-α7)_PDZ_ cluster still conserved. Clearly, the exact structural changes involved in the activation mechanism of HtrA2 remain open to debate until the crystal structure in the proteolytically active form is solved.

## Supporting Information

S1 Fig PCA of wtHtrA2/peptide trimer. Three most significant eigenvectors, modes 1–3, are represented as square displacements of sequential MD-time-averaged Cα coordinates. The secondary-structure elements of HtrA2 monomer (1LCY pdb entry) are indicated below the abscissa for reference. Clearly, the majority of segmental motions are explained in mode 1, as modes 2 and 3 contain only residual fluctuations of LB, L3 and the PD-PDZ linker. Unit A is in red, unit B green, unit C blue. The lower-bottom panel includes respective scree plots.
(PDF)

S2 Fig PCA of HtrA2S306A/peptide trimer. General legend see S1 Fig. Majority of segmental motions are explained in the first two modes, as mode 3 contains only residual fluctuations of LB, L3 and the PD-PDZ linker.
(PDF)

S3 Fig PCA of apo HtrA2S306A trimer. General legend see S1 Fig. Majority of segmental motions are explained in the first two modes, as mode 3 contains only residual fluctuations of LB, L3 and the PD-PDZ linker.
(PDF)

S4 Fig Visualization of motional modes 1–3 within unit C of wtHtrA2-peptide complex. Directional amplitudes (double-arrow modules ~ root squares of displacements in S1 Fig). of PCA-factorized motional modes of wtHtrA2-peptide complex are depicted on the HtrA2 mean structure. Cα-trace was interpolated to a smooth curve using VMD. The structures, in stereo, are oriented in agreement with all but top-right structures in Fig 5. Selected secondary-structure elements in mode 2 are marked. The motional arrows are not to the scale of decreasing variance (λ1/λ2/λ3 = 0.58/0.08/0.06). Instead, they are progressively scaled up by 0.87•(λ1-1/2, λ2-1/2, λ3-1/2), to visualize motions in modes 2 and 3. Double-arrows are cut off below 2Å, to expose only distinct segmental motions in each mode.
(PDF)

S1 File Supporting methods and results. (PDF)

S1 Table PCA of wtHtrA2/peptide trimer: the summary of the first 30 PCA modes. Factors 3 to 30 accumulate 48% to 85%, respectively, of total variance in Unit B, fair scree; and 73% to 93%, respectively, of total variance in Unit C, steep scree.
(PDF)

S2 Table PCA of HtrA2S306A/peptide trimer: the summary of the first 30 PCA modes. Factors 3 to 30 accumulate 67% to 91%, respectively, of total variance in Unit A, fair scree; and 81% to 96%, respectively, of total variance in Unit B, steep scree.
(PDF)

S3 Table PCA of apo HtrA2S306A trimer: the summary of the first 30 PCA modes. Factors 3 to 30 accumulate 49% to 88%, respectively, of total variance in Unit C, fair scree; and 69% to 91%, respectively, of total variance in Unit A, moderate scree.
(PDF)
