# Supplementary material for: Distinct 3D Architecture and Dynamics of the Human HtrA2(Omi) Protease and Its Mutated Variants
Source: PLoS One. 2016 Aug 29;11(8):e0161526. doi: 10.1371/journal.pone.0161526 (PMC5003398; doi:10.1371/journal.pone.0161526)
Supplement: S1 Fig — Three most significant eigenvectors, modes 1–3, are represented as square displacements of sequential MD-time-averaged Cα coordinates. The secondary-structure elements of HtrA2 monomer (1LCY pdb entry) are indicated below the abscissa for reference. Clearly, the majority of segmental motions are explained in mode 1, as modes 2 and 3 contain only residual fluctuations of LB, L3 and the PD-PDZ linker. Unit A is in red, unit B green, unit C blue. The lower-bottom panel includes respective scree plots. (PDF) [file pone.0161526.s001.pdf]

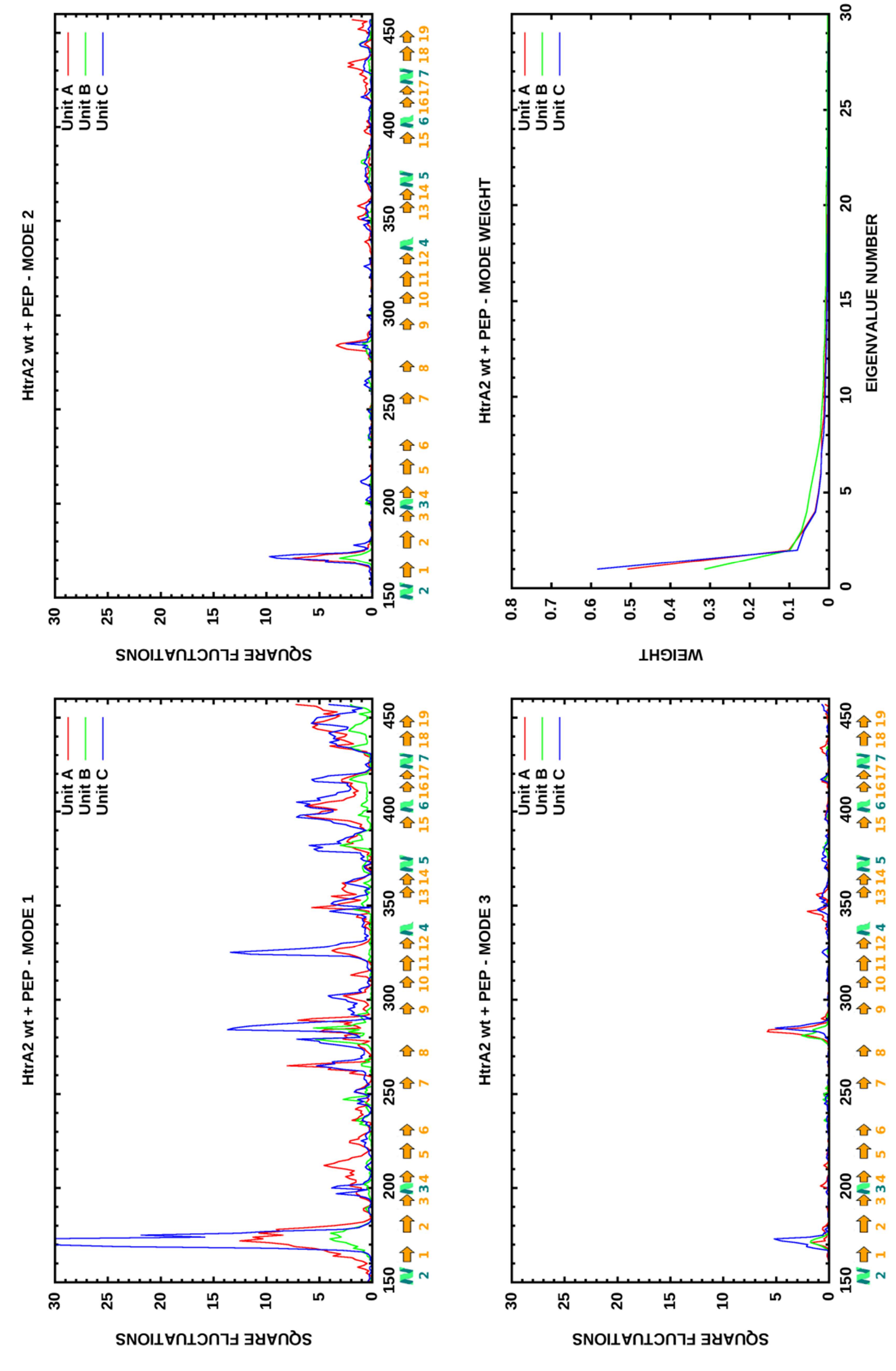

S1 Fig. PCA of wtHtrA2/peptide trimer. Three most significant eigenvectors, modes 1-3, are represented as square displacements of sequential MD-time-averaged Cα coordinates [1]. The secondary-structure elements of HtrA2 monomer (1LCY pdb entry [2]) are indicated below the abscissa for reference. Clearly, the majority of segmental motions are explained in mode 1, as modes 2 and 3 contain only residual fluctuations of LB, L3 and the PD-PDZ linker. Unit A is in red, unit B green, unit C blue. The lower-bottom panel includes respective scree plots.

References

1. *Yang LW, Eyal E, Bahar I, Kitao A. Principal component analysis of native ensembles of biomolecular structures (PCA_NEST): insights into functional dynamics. Bioinformatics. 2009;25(5):606-14. Epub 2009/01/17. doi: 10.1093/bioinformatics/btp023. PubMed PMID: 19147661; PubMed Central PMCID: PMC2647834.*
2. *Li W, Srinivasula SM, Chai J, Li P, Wu JW, Zhang Z, et al. Structural insights into the pro-apoptotic function of mitochondrial serine protease HtrA2/Omi. Nature structural biology. 2002;9(6):436-41. Epub 2002/04/23. doi: 10.1038/nsb795. PubMed PMID: 11967569.*
