# Supplementary material for: Distinct 3D Architecture and Dynamics of the Human HtrA2(Omi) Protease and Its Mutated Variants
Source: PLoS One. 2016 Aug 29;11(8):e0161526. doi: 10.1371/journal.pone.0161526 (PMC5003398; doi:10.1371/journal.pone.0161526)
Supplement: S2 Fig — General legend see S1 Fig. Majority of segmental motions are explained in the first two modes, as mode 3 contains only residual fluctuations of LB, L3 and the PD-PDZ linker. (PDF) [file pone.0161526.s002.pdf]

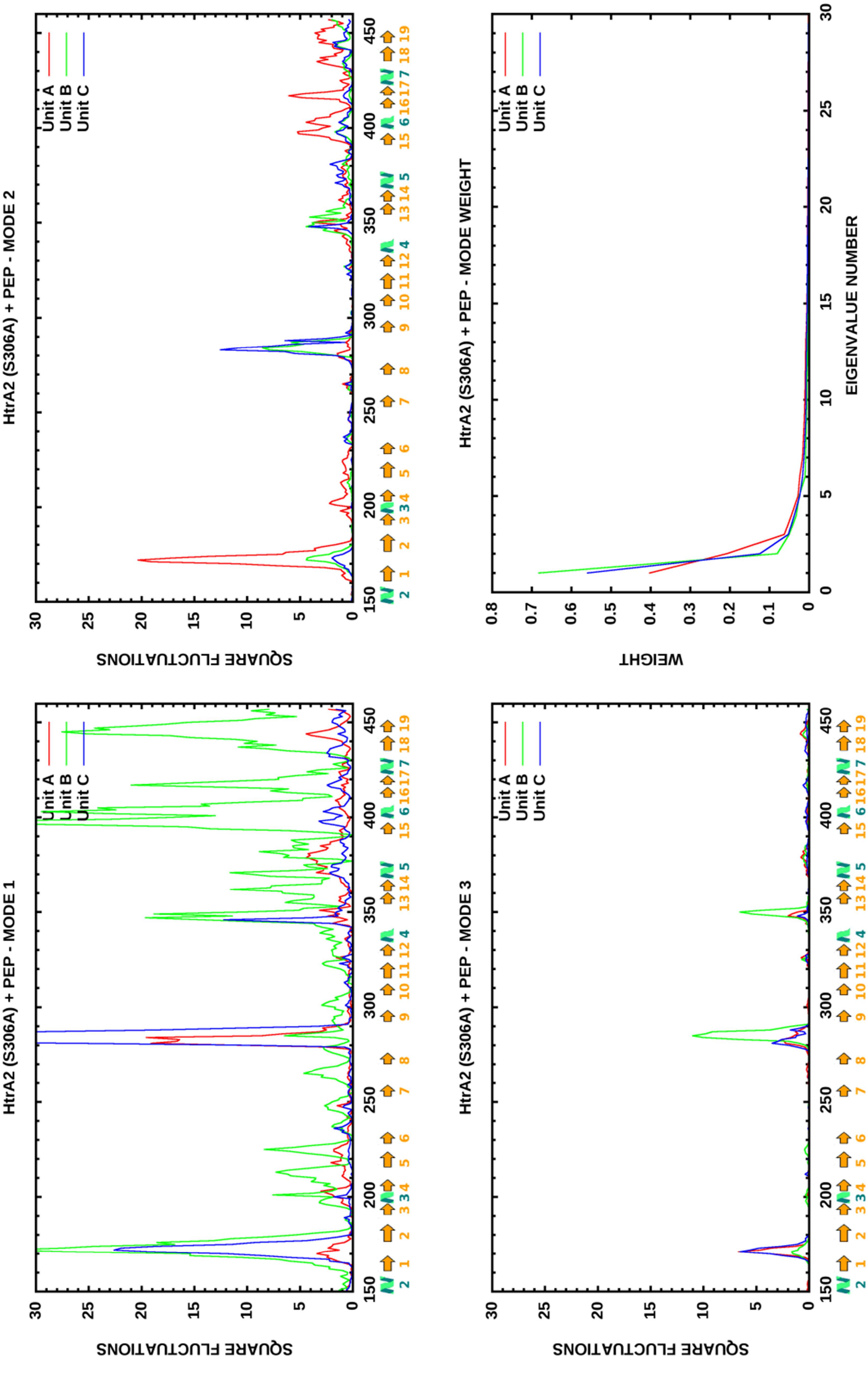

S2 Fig. PCA of HtrA2S306A/peptide trimer. General legend see S1 Fig. Majority of segmental motions are explained in the first two modes, as mode 3 contains only residual fluctuations of LB, L3 and the PD-PDZ linker.
