# Supplementary material for: Distinct 3D Architecture and Dynamics of the Human HtrA2(Omi) Protease and Its Mutated Variants
Source: PLoS One. 2016 Aug 29;11(8):e0161526. doi: 10.1371/journal.pone.0161526 (PMC5003398; doi:10.1371/journal.pone.0161526)
Supplement: S4 Fig — Directional amplitudes (double-arrow modules ~ root squares of displacements in S1 Fig). of PCA-factorized motional modes of wtHtrA2-peptide complex are depicted on the HtrA2 mean structure. Cα-trace was interpolated to a smooth curve using VMD. The structures, in stereo, are oriented in agreement with all but top-right structures in Fig 5. Selected secondary-structure elements in mode 2 are marked. The motional arrows are not to the scale of decreasing variance (λ1/λ2/λ3 = 0.58/0.08/0.06). Instead, they are progressively scaled up by 0.87•(λ1-1/2, λ2-1/2, λ3-1/2), to visualize motions in modes 2 and 3. Double-arrows are cut off below 2Å, to expose only distinct segmental motions in each mode. (PDF) [file pone.0161526.s004.pdf]

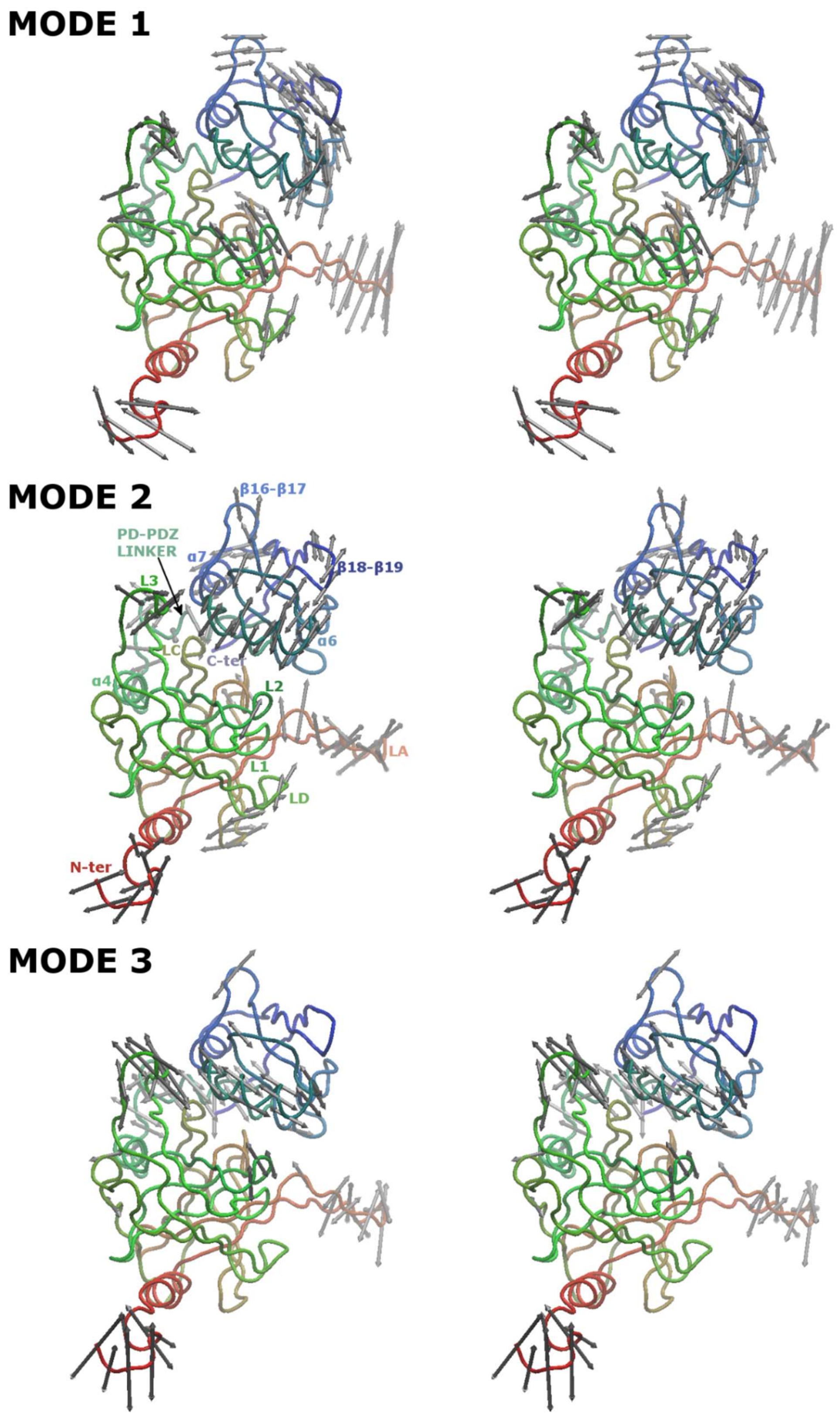

S4 Fig. Visualization of motional modes 1-3 within unit C of wtHtrA2-peptide complex. Directional amplitudes (double-arrow modules ~ root squares of displacements in S1 Fig.) of PCA-factorized motional modes of wtHtrA2-peptide complex are depicted on the HtrA2 mean structure. C^{α}-trace was interpolated to a smooth curve using VMD. The structures, in stereo, are oriented in agreement with all but top-right structures in Fig. 5. Selected secondary-structure elements in mode 2 are marked. The motional arrows are not to the scale of decreasing variance ($\lambda_1/\lambda_2/\lambda_3$=0.58/0.08/0.06). Instead, they are progressively scaled up by 0.87•($\lambda_1^{-1/2}$, $\lambda_2^{-1/2}$, $\lambda_3^{-1/2}$), to visualize motions in modes 2 and 3. Double-arrows are cut off below 2Å, to expose only distinct segmental motions in each mode.
