# Supplementary material for: Distinct 3D Architecture and Dynamics of the Human HtrA2(Omi) Protease and Its Mutated Variants
Source: PLoS One. 2016 Aug 29;11(8):e0161526. doi: 10.1371/journal.pone.0161526 (PMC5003398; doi:10.1371/journal.pone.0161526)
Supplement: S1 File — (PDF) [file pone.0161526.s005.pdf]

**Distinct 3D Architecture and Dynamics of the Human HtrA2(Omi) Protease and its Mutated Variants**

Artur Gieldon[1], Dorota Zurawa-Janicka[2], Miroslaw Jarzab[2], Tomasz Wenta[2], Przemyslaw Golik[3], Grzegorz Dubin[3,4,*], Barbara Lipinska[2], Jerzy Ciarkowski[1,*]

[1]Faculty of Chemistry, University of Gdansk, Wita Stwosza 63, 80-308 Gdansk, Poland

[2]Department of Biochemistry, Faculty of Biology, University of Gdansk, 80-308 Gdansk, Poland

[3]Faculty of Biochemistry, Biophysics and Biotechnology, Jagiellonian University, Gronostajowa 7, 30-387 Krakow, Poland

[4]Malopolska Centre of Biotechnology, ul. Gronostajowa 7a, 30-387 Krakow, Poland

*Corresponding authors

e-mail (GD): grzegorz.dubin@uj.edu.pl

e-mail (JC): jerzy.ciarkowski@ug.edu.pl

# Methods

## Principal Component Analysis (Essential Dynamics)

The Essential Dynamics introduced by Amadei et al. [1] turns up identical with the Principal Component Analysis, PCA, a statistical approach of a proven general use and efficiency [2]. The method is useful whenever major factors influencing/describing objects' behavior/properties have to be fished out from multitude of cross-correlated data [3, 4]. The method relies on diagonalization of an appropriately chosen covariance matrix (**Cov)**. To our aims, an MD trajectory matrix was initially translated to an origin, defined at the geometrical center of the protein (based only on $C^{\alpha}$ atom coordinates) and averaged over the MD-time. This gave ***MD*** matrix (size 3N · number of MD time-steps; N being the number of residues in a protein). **Cov** (of size 3Nx3N) resulted from multiplication of ***MD*** by its transpose $\boldsymbol{MD^T}$. **Cov,** when normalized, becomes identical with the respective correlation matrix. The diagonalization of **Cov**, i.e. solving an eigenproblem, leads to a set of 3N eigenvalues $\lambda_i$,(normalized $\lambda_i$/$\Sigma\lambda_i$ become weights; Σweights=1) and a 3Nx3N matrix of orthogonal eigenvectors, collectively with $\lambda_i$. The factors ordered by decreasing $\lambda_i$ signify declining major contributions of orthogonal (non-correlated) motional modes to the total dynamics of the protein. The effort turns out valuable when 3-4 major orthogonal modes suffice to explain a majority (70-80%, as measured by the sum of $\lambda_i$) of total mobility of the protein.

The PCA was performed using ProDy package [5]. Only Cα atom coordinates were processed (3x320 total; ligand ignored) and 5000 points from the productive MD trajectories (every 10 ps) were selected, resulting in the **Cov** size equal to 960x960. Analyses and visualizations of the PCA results were prepared using plug-ins to ProDy own NMWIZ routine and/or to the VMD [6] program.

# Results

## Principal Component Analysis

PCA confirms the observations of visual inspection of the MD results. Therefore, here we present only representative results on principal motional modes of HtrA2-ligand, HtrA2$^{S306A}$-ligand and apo HtrA2$^{S306A}$ (S1-S3 Tables and S1-S3 Figs., respectively). Scree plots (S1-S3 Figs.) clearly indicate rapid descents of the normalized eigenvalues. Remarkably, for units manifesting specific segmental/collective motions (units A and C in the HtrA2-ligand trimer and unit B in the HtrA2$^{S306A}$-ligand trimer; compare also Table 4) the scree plots are steeper than for the other ones. Indeed, for these three units, 70-80% of the total variance is explained in the first three factors (i.e. orthogonal collective/segmental motional modes), contrary to the other units, in which the first three factors typically explain only about 50-70% of the total motional variance, see S1-S3 Tables. The ratios of the eigenvalues (weights) of the first to the second factors ($\lambda_1/\lambda_2$) support this conclusion. They are noticeably larger ($\lambda_1/\lambda_2 \approx 5$-9) for the units experiencing specific movements than for the units moving more randomly ($\lambda_1/\lambda_2 \approx 2$-5). Steeper scree is associated with faster accumulation of variance through the first 30 factors (per total 3x320 $C^{\alpha}$ atoms, see Methods). For the units moving specifically, 90-95% of the total variance is included in the first 30 factors while for the others only 85-90%. The factors above around the 30$^{th}$ account for harmonic oscillations, Brownian motions and noise.

Three largest-eigenvalue factors (modes), evidently associated with segmental motions were analyzed in more detail. A bulk of segmental mobility of the HtrA2-ligand trimer is explained in the first factor (S1 Fig.), whereas the majority of segmental motions of the HtrA2$^{S306A}$-ligand and apo HtrA2$^{S306A}$ trimers are explained in the first two factors (S2 and S3 Figs.). Significant mobility of LA, L3 and the PD-PDZ-linker and lower than the average mobility of the inter-barrel linker and α7 are common for all HtrA2 models (S1-S3 Figs.). Mode 1 within units A and C of HtrA2-ligand trimer (S1 Fig.) and unit B of HtrA2$^{S306A}$-ligand trimer (S2 Fig.) describes these units' common segmental motion. This observation is

readily appreciable by comparison of the movements of linker and PDZ, especially if allowing for “mental scaling” between fluctuations of mode 1 in S1 and S2 Figs. For example, along with generally similar shapes of the segmental fluctuations in mode 1, in all three units α5 and α7 are in minimum while α6 and the β16-β17 and β18-β19 loops are in maximum. In S4 Fig., modes 1-3 of unit 3 of HtrA2-ligand-trimer are visualized as a representative example of a common motional behavior. Specifically, mode 1 (58,4% total variance) accounts for a major contribution of the segmental motion shown in Figure 5, referred to as a lid-opening (Fig. 5 and S4 Fig.). Mode 2 ( 8,0% total variance) accounts for another type of lid-opening, however, with the “joints-rotation” axis running horizontally and perpendicularly to that in mode 1 behind the PD-PDZ interface (Fig. 5 and S4 Fig.). Mode 3 (6,3% total variance) accounts for a minor unspecific motion of PDZ versus PD. As such, PCA results fully confirm all observations based solely on visual inspection of the MD results. Omnipresent superior LA, L3 and linker mobility (see above) is also reflected in S4 Fig.

# References


1. Amadei A, Linssen AB, Berendsen HJ. Essential dynamics of proteins. Proteins. 1993;17(4):412-25. Epub 1993/12/01. doi: 10.1002/prot.340170408. PubMed PMID: 8108382.

2. Yang LW, Eyal E, Bahar I, Kitao A. Principal component analysis of native ensembles of biomolecular structures (PCA_NEST): insights into functional dynamics. Bioinformatics. 2009;25(5):606-14. Epub 2009/01/17. doi: 10.1093/bioinformatics/btp023. PubMed PMID: 19147661; PubMed Central PMCID: PMC2647834.

3. Lawley DN, Maxwell AE. Factor Analysis as a Statistical Method. 2nd ed, Butterworths, London. 1971.

4. Malinowski E, Hovery DG. Factor Analysis in Chemistry. Wiley, New York. 1980.

5. Bakan A, Meireles LM, Bahar I. ProDy: protein dynamics inferred from theory and experiments. Bioinformatics. 2011;27(11):1575-7. Epub 2011/04/08. doi: 10.1093/bioinformatics/btr168. PubMed PMID: 21471012; PubMed Central PMCID: PMC3102222.

6. Humphrey W, Dalke A, Schulten K. VMD: visual molecular dynamics. Journal of molecular graphics. 1996;14(1):33-8, 27-8. Epub 1996/02/01. PubMed PMID: 8744570.
