# Supplementary material for: Distinct 3D Architecture and Dynamics of the Human HtrA2(Omi) Protease and Its Mutated Variants
Source: PLoS One. 2016 Aug 29;11(8):e0161526. doi: 10.1371/journal.pone.0161526 (PMC5003398; doi:10.1371/journal.pone.0161526)
Supplement: S1 Table — Factors 3 to 30 accumulate 48% to 85%, respectively, of total variance in Unit B, fair scree; and 73% to 93%, respectively, of total variance in Unit C, steep scree. (PDF) [file pone.0161526.s006.pdf]

S1 Table. PCA of wtHtrA2/peptide trimer: the summary of the first 30 PCA modes. Factors 3 to 30 accumulate 48% to 85%, respectively, of total variance in Unit B, fair scree; and 73% to 93%, respectively, of total variance in Unit C, steep scree.

	wtHtrA2-ligand Unit A		wtHtrA2-ligand Unit B		wtHtrA2-ligand Unit C	
**Mode No**	**Eigenvalue/ Factor Weight**	**Cumulative variance**	**Eigenvalue/ Factor Weight**	**Cumulative variance**	**Eigenvalue/ Factor Weight**	**Cumulative variance**
1	0,5070	0,5070	0,3126	0,3126	0,5836	0,5836
2	0,1018	0,6088	0,0989	0,4115	0,0796	0,6631
3	0,0663	0,6751	0,0700	0,4814	0,0630	0,7262
4	0,0352	0,7103	0,0564	0,5379	0,0341	0,7602
5	0,0263	0,7366	0,0482	0,5860	0,0260	0,7862
6	0,0211	0,7576	0,0389	0,6249	0,0201	0,8063
7	0,0189	0,7766	0,0298	0,6546	0,0197	0,8260
8	0,0185	0,7951	0,0217	0,6763	0,0140	0,8400
9	0,0132	0,8083	0,0193	0,6955	0,0111	0,8510
10	0,0122	0,8204	0,0158	0,7113	0,0091	0,8601
11	0,0104	0,8308	0,0139	0,7252	0,0087	0,8688
12	0,0094	0,8403	0,0135	0,7387	0,0071	0,8760
13	0,0077	0,8480	0,0109	0,7496	0,0065	0,8825
14	0,0065	0,8544	0,0099	0,7595	0,0059	0,8884
15	0,0064	0,8608	0,0093	0,7689	0,0050	0,8934
16	0,0052	0,8661	0,0082	0,7770	0,0044	0,8978
17	0,0047	0,8708	0,0079	0,7850	0,0041	0,9019
18	0,0043	0,8751	0,0073	0,7923	0,0037	0,9055
19	0,0043	0,8794	0,0070	0,7992	0,0030	0,9086
20	0,0036	0,8830	0,0069	0,8062	0,0029	0,9115
21	0,0034	0,8864	0,0062	0,8124	0,0026	0,9141
22	0,0032	0,8896	0,0054	0,8178	0,0026	0,9167
23	0,0031	0,8927	0,0051	0,8228	0,0024	0,9191
24	0,0030	0,8957	0,0046	0,8274	0,0022	0,9213
25	0,0026	0,8984	0,0044	0,8317	0,0021	0,9234
26	0,0025	0,9008	0,0042	0,8359	0,0020	0,9254
27	0,0025	0,9033	0,0041	0,8401	0,0019	0,9273
28	0,0024	0,9057	0,0039	0,8440	0,0018	0,9291
29	0,0022	0,9079	0,0037	0,8477	0,0016	0,9307
30	0,0021	0,9100	0,0035	0,8512	0,0016	0,9323
