# Supplementary material for: Distinct 3D Architecture and Dynamics of the Human HtrA2(Omi) Protease and Its Mutated Variants
Source: PLoS One. 2016 Aug 29;11(8):e0161526. doi: 10.1371/journal.pone.0161526 (PMC5003398; doi:10.1371/journal.pone.0161526)
Supplement: S2 Table — Factors 3 to 30 accumulate 67% to 91%, respectively, of total variance in Unit A, fair scree; and 81% to 96%, respectively, of total variance in Unit B, steep scree. (PDF) [file pone.0161526.s007.pdf]

**Table S2. PCA of HtrA2$^{S306A}$/peptide trimer**: the summary of the first 30 PCA modes. Factors 3 to 30 accumulate 67% to 91%, respectively, of total variance in Unit A, fair scree; and 81% to 96%, respectively, of total variance in Unit B, steep scree.

| | HtrA2$^{S306A}$-ligand Unit A | | HtrA2$^{S306A}$ -ligand Unit B | | HtrA2$^{S306A}$ -ligand Unit C | |
|---|---|---|---|---|---|---|
| **Mode No** | **Eigenvalue/ Factor Weight** | **Cumulative variance** | **Eigenvalue/ Factor Weight** | **Cumulative variance** | **Eigenvalue/ Factor Weight** | **Cumulative variance** |
| 1 | 0,4025 | 0,4025 | 0,6824 | 0,6824 | 0,5595 | 0,5595 |
| 2 | 0,2081 | 0,6106 | 0,0804 | 0,7628 | 0,1245 | 0,6840 |
| 3 | 0,0624 | 0,6729 | 0,0505 | 0,8132 | 0,0526 | 0,7366 |
| 4 | 0,0445 | 0,7174 | 0,0327 | 0,8459 | 0,0376 | 0,7742 |
| 5 | 0,0280 | 0,7454 | 0,0254 | 0,8713 | 0,0239 | 0,7981 |
| 6 | 0,0240 | 0,7694 | 0,0100 | 0,8813 | 0,0163 | 0,8144 |
| 7 | 0,0164 | 0,7858 | 0,0084 | 0,8897 | 0,0133 | 0,8276 |
| 8 | 0,0147 | 0,8005 | 0,0078 | 0,8975 | 0,0105 | 0,8381 |
| 9 | 0,0122 | 0,8126 | 0,0064 | 0,9039 | 0,0084 | 0,8465 |
| 10 | 0,0107 | 0,8234 | 0,0057 | 0,9096 | 0,0080 | 0,8545 |
| 11 | 0,0102 | 0,8336 | 0,0055 | 0,9151 | 0,0079 | 0,8624 |
| 12 | 0,0093 | 0,8429 | 0,0042 | 0,9194 | 0,0072 | 0,8696 |
| 13 | 0,0089 | 0,8518 | 0,0039 | 0,9232 | 0,0062 | 0,8758 |
| 14 | 0,0068 | 0,8586 | 0,0034 | 0,9267 | 0,0054 | 0,8813 |
| 15 | 0,0059 | 0,8645 | 0,0033 | 0,9299 | 0,0051 | 0,8864 |
| 16 | 0,0050 | 0,8695 | 0,0030 | 0,9330 | 0,0047 | 0,8911 |
| 17 | 0,0044 | 0,8740 | 0,0026 | 0,9355 | 0,0045 | 0,8956 |
| 18 | 0,0042 | 0,8781 | 0,0022 | 0,9378 | 0,0043 | 0,8999 |
| 19 | 0,0041 | 0,8822 | 0,0021 | 0,9398 | 0,0033 | 0,9032 |
| 20 | 0,0036 | 0,8858 | 0,0021 | 0,9419 | 0,0032 | 0,9064 |
| 21 | 0,0035 | 0,8894 | 0,0019 | 0,9438 | 0,0029 | 0,9093 |
| 22 | 0,0032 | 0,8926 | 0,0017 | 0,9455 | 0,0027 | 0,9120 |
| 23 | 0,0030 | 0,8956 | 0,0017 | 0,9472 | 0,0025 | 0,9144 |
| 24 | 0,0029 | 0,8986 | 0,0015 | 0,9486 | 0,0023 | 0,9167 |
| 25 | 0,0029 | 0,9015 | 0,0014 | 0,9500 | 0,0022 | 0,9189 |
| 26 | 0,0029 | 0,9043 | 0,0013 | 0,9514 | 0,0021 | 0,9210 |
| 27 | 0,0026 | 0,9069 | 0,0013 | 0,9526 | 0,0020 | 0,9230 |
| 28 | 0,0023 | 0,9092 | 0,0012 | 0,9538 | 0,0019 | 0,9249 |
| 29 | 0,0022 | 0,9114 | 0,0011 | 0,9549 | 0,0018 | 0,9267 |
| 30 | 0,0020 | 0,9134 | 0,0011 | 0,9560 | 0,0017 | 0,9284 |
