# Supplementary material for: Distinct 3D Architecture and Dynamics of the Human HtrA2(Omi) Protease and Its Mutated Variants
Source: PLoS One. 2016 Aug 29;11(8):e0161526. doi: 10.1371/journal.pone.0161526 (PMC5003398; doi:10.1371/journal.pone.0161526)
Supplement: S3 Table — Factors 3 to 30 accumulate 49% to 88%, respectively, of total variance in Unit C, fair scree; and 69% to 91%, respectively, of total variance in Unit A, moderate scree. (PDF) [file pone.0161526.s008.pdf]

S3 Table. PCA of apo HtrA2^{S306A} trimer: the summary of the first 30 PCA modes. Factors 3 to 30 accumulate 49% to 88%, respectively, of total variance in Unit C, fair scree; and 69% to 91%, respectively, of total variance in Unit A, moderate scree.

	HtrA2^{S306A} Unit A		HtrA2^{S306A} Unit B		HtrA2^{S306A} Unit C	
**Mode No**	**Eigenvalue/ Factor Weight**	**Cumulative variance**	**Eigenvalue/ Factor Weight**	**Cumulative variance**	**Eigenvalue/ Factor Weight**	**Cumulative variance**
1	0,4640	0,4640	0,4418	0,4418	0,3144	0,3144
2	0,1845	0,6485	0,0934	0,5352	0,0956	0,4100
3	0,0454	0,6939	0,0513	0,5865	0,0782	0,4882
4	0,0308	0,7247	0,0419	0,6285	0,0689	0,5572
5	0,0272	0,7519	0,0373	0,6657	0,0551	0,6123
6	0,0203	0,7722	0,0338	0,6995	0,0369	0,6492
7	0,0182	0,7905	0,0278	0,7274	0,0297	0,6788
8	0,0154	0,8059	0,0209	0,7483	0,0248	0,7037
9	0,0119	0,8178	0,0188	0,7671	0,0239	0,7275
10	0,0104	0,8281	0,0166	0,7837	0,0186	0,7462
11	0,0084	0,8365	0,0112	0,7949	0,0176	0,7638
12	0,0079	0,8444	0,0092	0,8040	0,0127	0,7764
13	0,0078	0,8522	0,0085	0,8125	0,0116	0,7880
14	0,0066	0,8588	0,0079	0,8204	0,0091	0,7972
15	0,0054	0,8642	0,0069	0,8273	0,0086	0,8058
16	0,0049	0,8692	0,0062	0,8335	0,0079	0,8136
17	0,0048	0,8740	0,0056	0,8391	0,0069	0,8205
18	0,0044	0,8784	0,0054	0,8445	0,0063	0,8268
19	0,0044	0,8828	0,0052	0,8497	0,0062	0,8330
20	0,0038	0,8866	0,0046	0,8543	0,0051	0,8381
21	0,0036	0,8902	0,0042	0,8585	0,0049	0,8430
22	0,0033	0,8935	0,0038	0,8624	0,0045	0,8475
23	0,0031	0,8965	0,0037	0,8661	0,0043	0,8519
24	0,0029	0,8994	0,0034	0,8696	0,0041	0,8559
25	0,0027	0,9021	0,0033	0,8729	0,0038	0,8597
26	0,0025	0,9047	0,0033	0,8761	0,0035	0,8632
27	0,0024	0,9071	0,0031	0,8793	0,0032	0,8664
28	0,0022	0,9092	0,0030	0,8823	0,0030	0,8694
29	0,0021	0,9113	0,0029	0,8852	0,0029	0,8723
30	0,0021	0,9134	0,0028	0,8880	0,0028	0,8751
